# Unique Applications of *para*-Hydrogen Matrix Isolation to Spectroscopy and Astrochemistry

**DOI:** 10.1021/acs.jpclett.4c02733

**Published:** 2024-11-06

**Authors:** Isabelle Weber, Prasad Ramesh Joshi, David T. Anderson, Yuan-Pern Lee

**Affiliations:** † Department of Applied Chemistry and Institute of Molecular Science, National Yang Ming Chiao Tung University, Hsinchu 300093, Taiwan; ‡ Department of Chemistry, University of Wyoming, Laramie, Wyoming 82071, United States; § Center for Emergent Functional Matter Science, National Yang Ming Chiao Tung University, Hsinchu 300093, Taiwan

## Abstract

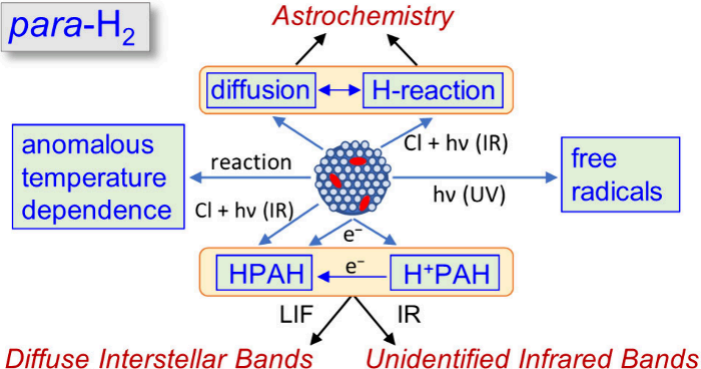

Cryogenic solid *para*-hydrogen (*p*-H_2_) exhibits pronounced quantum effects, enabling unique experiments that are typically not possible in noble-gas matrices. The diminished cage effect facilitates the production of free radicals via *in situ* photolysis or photoinduced reactions. Electron bombardment during deposition readily produces protonated and hydrogenated species, such as polycyclic aromatic hydrocarbons, that are important in astrochemistry. In addition, quantum diffusion delocalizes hydrogen atoms in solid *p*-H_2_, allowing efficient H atom reactions with astrochemical species and introducing new concepts in astrochemical models. Some H atom reactions display anomalous temperature behaviors, highlighting the rich chemistry in *p*-H_2_. The investigation on quantum diffusion of heavier atoms and molecules is also important for our understanding of the chemistry in interstellar ice. Additionally, matrix shifts of electronic transitions of polycyclic aromatic hydrocarbons in *p*-H_2_ are less divergent than those in solid Ne such that systematic measurements in *p*-H_2_ might help in the assignment of diffuse interstellar bands.

The matrix-isolation technique using noble-gas matrix hosts (Ne, Ar, Kr, Xe, and N_2_) has been widely employed to preserve unstable species for spectroscopic or chemical studies.^1−5^ However, because of the cage effect associated with the rigid crystal structure of the matrix host, to produce free radicals via *in situ* photolysis or photoinduced reactions is challenging. For the past several decades, *para*-hydrogen (*p*-H_2_) has emerged as a novel matrix host due to its unique properties associated with its quantum-mechanical nature.^6^ Initially, focus was placed on developing experimental techniques to grow transparent, chemically doped *p*-H_2_ solids, investigating the unique properties of solid *p*-H_2_, performing high-resolution solid-state spectroscopy of molecules and clusters, producing free radicals via *in situ* photolysis, and studying nuclear-spin relaxation with infrared spectroscopy; several earlier reviews are available.^5−10^ In more recent years, more and more applications that are unique to *p*-H_2_, including using *in situ* photolysis and photoinduced reactions to produce radicals, using electron bombardment to produce protonated and hydrogenated species, and making use of the H atom mobility in solid *p*-H_2_ to carry out low-temperature hydrogen tunneling reactions, have been developed, as summarized in several articles.^11−14^ This Perspective will hence focus on the more recent advances on photoinduced fragmentation and reactions, production of protonated species, H atom reactions, anomalous temperature effects in chemical reactivity, heavy-atom diffusion, and electronic transitions of trapped guest species that are unique to *p*-H_2_ matrix-isolation spectroscopy; the last three topics were not covered in detail in previous reviews. Possible applications to astrochemistry and future perspectives are also discussed. Due to limited space, the nuclear-spin relaxation of species in *p*-H_2_ is not included in this article.

## Photolysis and Photoinduced Reactions *In Situ*

The advantage of the diminished cage effect in solid *p*-H_2_ for producing free radicals was first demonstrated by Momose and co-workers to produce ^•^CH_3_ and ^•^C_2_H_5_ upon UV irradiation of CH_3_I and C_2_H_5_I trapped in solid *p*-H_2_.^7,15^ Later, Lee and co-workers demonstrated that CH_3_^•^S and CH_3_•O can be readily produced by photolysis of CH_3_SSCH_3_ (or CH_3_SCH_3_)^16^ and CH_3_ONO,^17^ respectively; whereas similar experiments in solid Ar produced only H_2_CS + CH_3_SH and H_2_CO + HNO because the solid Ar solvent cage constrained the two nascent fragments so that they reacted further with each other to form stable molecules. With the diminished cage effect, producing free radicals from *in situ* photolysis becomes straightforward in solid *p*-H_2_; for example, many alkyl radicals can be readily produced from UV irradiation of alkyl halides.^18^ More recently, (*Z*)- and (*E*)-C_2_H_3_^•^C(CH_3_)I radicals were produced upon *in situ* photodissociation of (*Z*)- and (*E*)-(CH_2_I)HC=C(CH_3_)I, respectively,^19^ and (*Z*)- and (*E*)-^•^CH_2_C(CH_3_)CHI radicals were observed upon photodissociation of (*Z*)- and (*E*)-(CH_2_I)(CH_3_)C=CHI, respectively;^20^ these molecules are precursors of Criegee intermediates. The allylic C–I bond, not the vinylic one, was dissociated, and the (*Z*)- and (*E*)-conformation was retained upon photolysis. Momose and co-workers employed UV or vacuum UV irradiation (193, 213, or 266 nm) to photolyze 1,3-cyclohexadiene^21^ and several amino acids including α-alanine,^22^ glycine, leucine, proline, and serine^23^ to observe HOCO and imines rather than amine radicals; they proposed that HOCO could be a tracer for the search of amino acids in interstellar space.

The diminished cage effect also makes photoinduced bimolecular reactions feasible in solid *p*-H_2_. A series of reactions of halogen atoms with various alkenes to produce the haloalkyl radicals have been demonstrated.^14^ For example, photolysis of a Cl_2_/isoprene/*p*-H_2_ matrix produced 1-chloromethyl-1-methylallyl and 1-chloromethyl-2-methylallyl radicals, showing site selectivity on the addition of the Cl atom to the carbon skeleton;^24^ in contrast, only stable dichloroalkanes were produced from analogous experiments in an Ar matrix. Formation and the infrared spectrum of the open-form of the 2-bromoethyl radical (2-^•^C_2_H_4_Br) from UV irradiation of a C_2_H_4_/Br_2_/*p*-H_2_ matrix were also reported.^25^ This feature of a diminished cage effect in *p*-H_2_ matrix isolation opens a new paradigm for producing free radicals that were challenging to prepare either in the gaseous phase or in noble-gas matrices.

**Figure 1:**
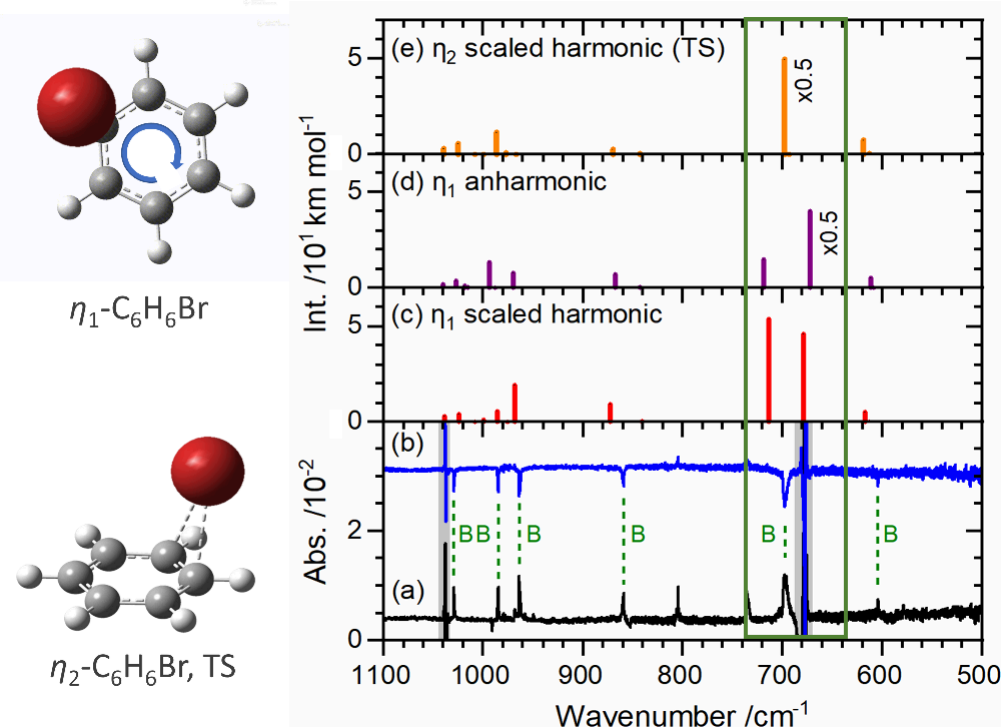
Bevel-gear-type motion of •C_6_H_6_Br and its infrared spectrum. The benzene ring undergoes a bevel-gear-type motion with respect to Br in η_1_-^•^C_6_H_6_Br. η_2_-^•^C_6_H_6_Br is a transition state with a barrier of ∼1 kJ mol^–1^. Spectral lines indicated with B (green) are due to η_1_-^•^C_6_H_6_Br. In the green box, two lines of out-of-plane CH bending modes were predicted for η_1_-^•^C_6_H_6_Br, but only one broad feature was observed. Partially reproduced from ref (27). Copyright 2023 American Chemical Society.

In addition to the production of unstable species, the properties of weakly bound radicals in solid *p*-H_2_ provide insight into the nature of the reaction. Unlike the ^•^C_6_H_6_Cl, an open-form η_1_ σ-complex (6-chlorocyclohexadienyl radical) reported previously in solid *p*-H_2_,^26^ the ^•^C_6_H_6_Br radical produced via *in situ* photolysis of a Br_2_/C_6_H_6_/*p*-H_2_ matrix is an open-form η_1_ π-complex with the benzene ring performing a bevel-gear-type rotation (Figure 1) with respect to Br, according to its IR absorption spectrum that shows the two predicted CH out-of-plane bending modes associated with mainly even- and odd-numbered carbons merged into one broad band; this motion was also supported by quantum-chemical computations showing a small barrier ∼1 kJ mol^–1^.^27^ Furthermore, the observation of only *trans*-*ortho*- and *trans*-*para*-C_6_H_6_Br_2_, but not *cis*-*ortho*- and *cis*-*para*-C_6_H_6_Br_2_, when the mixing ratio of Br_2_ was increased suggests that this gear-type motion allows the second Br atom to attack the ^•^C_6_H_6_Br radical from only the opposite side of the Br atom in ^•^C_6_H_6_Br. Previously, it was generally accepted that this stereo selectivity was associated with the formation of an η_2_ complex, with Br bound to two carbon atoms, even though no direct detection of the η_2_ complex has been reported. This finding indicates that an η_1_ complex with a bevel-gear-type motion, which might also be feasible in solutions, can explain this stereo selectivity.

One of the chief limitations to using solid *p*-H_2_ as a matrix host to generate free radicals or ions for spectroscopic characterization is the fact that H_2_ is not chemically inert, as pointed out previously;^11^ examples include reactions of radicals ^•^CH_2_, ^•^C_2_H_3_, and ^•^C_2_H_2_Cl, produced via photolysis or photoinduced reactions, with the H_2_ host, so that these radicals could not be isolated in *p*-H_2_. However, while H_2_ molecules are obviously more reactive than noble gas atoms, the large bond energy of H_2_ (436 kJ mol^–1^) combined with the high thermal conductivity of *p*-H_2_ crystals^7^ makes solid *p*-H_2_ relatively chemically inert. Nonetheless, if the target reactive species can react with the *p*-H_2_ host via an exergonic reaction, then even if the barrier to reaction is very high, the possibility of tunneling reactions with the host can prevent stabilization of the target species at enough concentrations to detect it. Sometimes if the tunneling reaction with the surrounding hydrogen molecules is on a time scale of a few hours, the reactive species can still be observed by conventional spectroscopy, and this disadvantage can be turned into an advantage. For example, infrared studies of the *in situ* photodissociation of NH_3_ in solid *p*-H_2_ at 193 nm showed that the nascent-^•^NH_2_ photoproduct is rapidly cooled within the *p*-H_2_ matrix to the ground vibrational and rotational state before a tunneling-driven reaction (τ_1/2_= 0.37 min, *E*_A_ = 47.5 kJ mol^–1^) with the *p*-H_2_ host to produce *ortho*-NH_3_ in a defect site.^28^

## Protonated and Hydrogenated PAH

Electron bombardment during deposition of a *p*-H_2_ matrix produces a significant amount of H_3_^+^ and H atoms from the reaction H_2_^+^ + H_2_; H_3_^+^ can readily transfer a proton to a species that has a proton affinity greater than that (422 kJ mol^–1^) of H_2_. Most polycyclic aromatic hydrocarbons (PAH) have proton affinities greater than 800 kJ mol^–1^, and therefore the production of protonated PAH (H^+^PAH) via proton transfer from H_3_^+^ becomes feasible. The side products are hydrogenated PAH, which can be produced from either the neutralization of a protonated PAH or the reaction of an H atom with the PAH. In general, this method has advantages over Ar-tagging or IR multiphoton dissociation methods for recording the IR spectrum of cold ions;^11^ among them, the FTIR absorption spectrum has a wide spectral coverage and provides accurate relative IR intensities for better comparison with theoretical predictions. Also, because guest ions or molecules typically have narrow absorption line widths in *p*-H_2_, spectra of various isomers can be resolved and identified by grouping observed lines of each isomer according to their distinct photolytic behaviors upon secondary photolysis at varied wavelengths. The protonation of aniline (C_6_H_5_NH_2_) using this technique serves as a good example; *para*-, *amino*-, and *ortho*-H^+^C_6_H_5_NH_2_ isomers were clearly identified according to their IR spectra.^29^ Similarly, isomers of protonated fluoranthene, 3-, 9-, and 10-C_16_H_11_^+^, were all identified with IR spectra in a single experiment.^30^

Protonated PAH were proposed to be possible carriers of the interstellar unidentified infrared (UIR) emission bands.^31^ Earlier work on planar protonated PAH isolated in *p*-H_2_ with increasing size from naphthalene (1-C_10_H_9_^+^),^32^ pyrene (1-C_16_H_11_^+^),^33^ coronene (1-C_24_H_13_^+^),^34^ to ovalene (7-C_32_H_15_^+^)^35^ indicated that, as the size of PAH increases, the IR absorption bands are trending toward, but not quite reaching, the UIR emission bands.^11,14^ Furthermore, the IR absorption bands of protonated corannulene (*hub*-H^+^C_20_H_10_),^36^ a nonplanar PAH which has a carbon framework as a fragment of C_60_, also agree satisfactorily with those of the UIR bands. More recent work focused on polycyclic aromatic nitrogen heterocycles (PANH). The N atom in the aromatic ring of protonated PANH (H^+^PANH), such as protonated quinoline (C_9_H_7_NH^+^)^37^ and protonated isoquinoline (*iso*-C_9_H_7_NH^+^),^38^ induces a blue shift of the CC-stretching modes of PAH near 6.3 μm, so that these bands match better with the UIR band near 6.2 μm. However, other bands of C_9_H_7_NH^+^ and *iso*-C_9_H_7_NH^+^ do not fit with the UIR bands, suggesting that these two species are unlikely to be carriers of the UIR bands, consistent with the expectation that they are too small to survive the intense UV fields in interstellar media.

The associated coproducts monohydrogenated PANH (HPANH) were produced either from the neutralization of the protonated H^+^PANH or reactions of H with PANH in the electron-bombardment experiments. These HPANH also can be produced by a more efficient method via UV/IR irradiation of the *p*-H_2_ matrix with trace Cl_2_ (typically <200 ppm) added to produce H atoms via Cl + H_2_ (*v* = 1), to be discussed in the next section. Because of the small mixing ratio of H atoms employed in these experiments, mainly only monohydrogenated PAH or PANH were produced. In general, PAH/PANH hydrogenated at various nonfused carbon sites have similar energy, so that many isomers might be produced. These isomers were distinguished according to their photolytic behavior upon irradiation at varied wavelengths and by comparison with quantum-chemically predicted IR spectra. For quinoline (C_9_H_7_N), hydrogenation at the 1-, 3-, 4-, 7-, and 8-positions was observed,^37^ whereas for isoquinoline (*iso*-C_9_H_7_N), hydrogenation at all eight feasible carbon sites except the two carbons on the fused ring was observed.^38^ According to the CCSD(T)/6-311++G(d,p)//B3LYP/6-311++G(d,p) method, the lowest-energy isomer of *iso*-^•^C_9_H_8_N is 2-*iso*-^•^C_9_H_8_N (or *iso*-^•^C_9_H_7_NH, hydrogenation at the N site), other isomers with hydrogenation on the nonfused carbon sites are higher in energy by only 2–24 kJ mol^–1^; the barriers for the hydrogenation range from 21 to 27 kJ mol^–1^, so they are all accessible from H atom tunneling reactions. Some missing isomers in hydrogenated quinoline might be due to interference from other species or their small IR intensities. This technique is unique in producing nearly all possible monohydrogenated PAH in a single experiment.

## Hydrogen Atom Reactions

**Figure 2:**
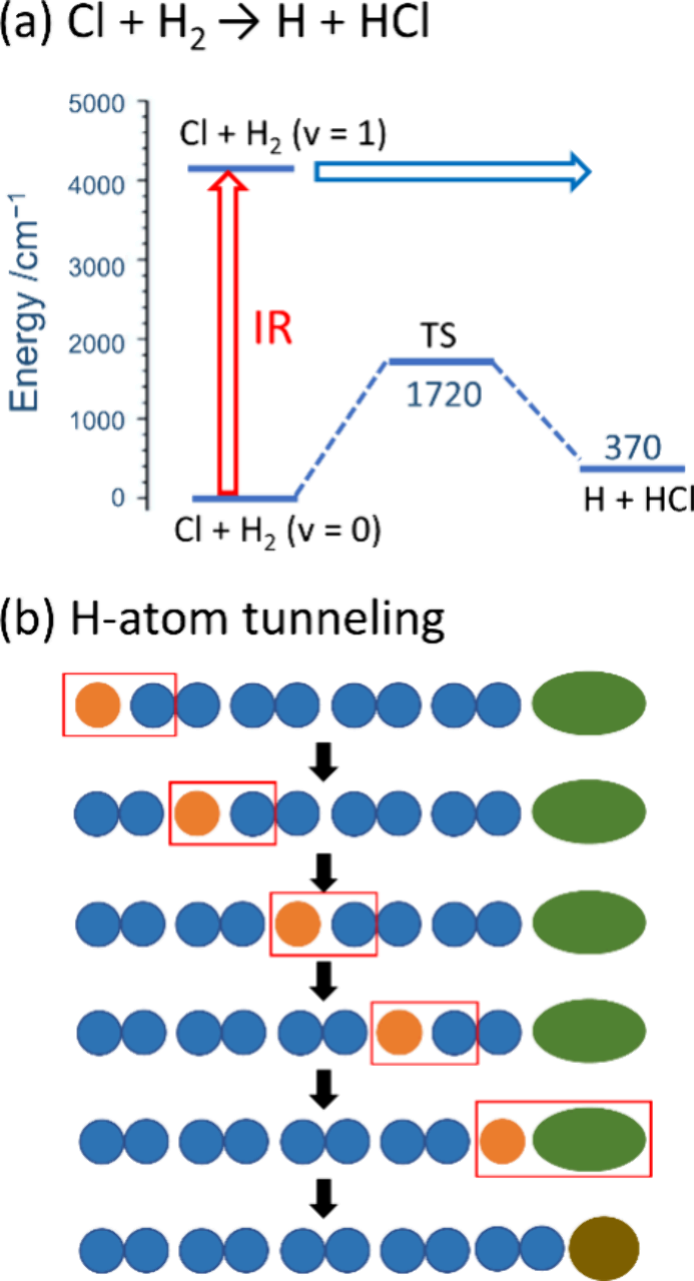
H atom tunneling reaction in solid *p*-H_2_. (a) Potential energy scheme of Cl + H_2_. The reaction is endothermic by ∼4 kJ mol^–1^ (370 cm^–1^) with a barrier of ∼21 kJ mol^–1^ (1720 cm^–1^). The reaction proceeds upon IR irradiation. (b) H atom tunneling in the solid *p*-H_2_. The pair of blue balls indicates H_2_, and the orange, green, and brown balls indicate H atom, reactant, and product, respectively. The red box indicates the formation of a new bond. The H atom does not have to diffuse physically through the matrix to approach the reactant.

One convenient method used to generate H atoms in solid *p*-H_2_ is to take advantage of the diminished cage effect to photolyze trace Cl_2_ (<1000 ppm) to form isolated Cl atoms; Cl atoms are stable in *p*-H_2_ because the reaction Cl + H_2_ is endothermic by ∼4 kJ mol^–1^ (∼370 cm^–1^) with a barrier of ∼21 kJ mol^–1^ (∼1720 cm^–1^).^13^ Next, to generate the H atoms, the matrix is irradiated with near-IR radiation which is absorbed by the *p*-H_2_ matrix in the 4200–4700 cm^–1^ range and generates H_2_(*v* = 1) vibrons that travel through the solid and induce the Cl + H_2_(*v* = 1) reaction (Figure 2a). Using this UV/IR method, the H atom is initially produced with some fraction of the exothermicity (∼45.3 kJ mol^–1^) of the Cl + H_2_(*v* = 1) reaction; however, this energy is quickly dissipated by the *p*-H_2_ matrix, and the H atom is rapidly thermalized.^39^ Once the H atom is generated in solid *p*-H_2_ it becomes delocalized by a chemical diffusion mechanism (H + H_2_ → H_2_ + H) whereby the H atom reacts repeatedly with adjacent H_2_ molecules (Figure 2b) and in this way moves through the solid.^40^ Typically, researchers that use this UV/IR method to generate H atoms also perform experiments in the dark to test that the reaction still occurs, even in the absence of any near-IR radiation. By studying the reaction both during the near-IR exposure and in the dark, researchers gain further insight into the reaction mechanism. Alternatively, the group in Laramie employed 193 nm irradiation of N_2_O to produce O(^1^D), which reacted with the surrounding H_2_ host to produce H atoms (along with OH); reaction of H atoms with N_2_O produced both *cis*- and *trans*-HNNO.^41^

Reactions of H atoms play key roles in astrochemical models for the formation of complex organic molecules because H atoms are abundant, mobile, and reactive. A detailed model for the formation of H_2_O, H_2_CO, and CH_3_OH involving H-abstraction and H-addition reactions has been reported;^42^ for example, H atom addition can convert CO sequentially to H*CO, H_2_CO, CH_3_*O or *CH_2_OH, and CH_3_OH.^42−44^ However, many astrochemical models typically focus on mainly H atom addition reactions.^45,46^ For H atom addition reactions, infrared spectra of *cis*- and *trans*-HNNO from H + N_2_O;^41^ 1-^•^C_4_H_7_ from H + 1,3-butadiene (C_4_H_6_);^47^ 1,1-dimethylallyl [(CH_3_)_2_^•^CCH=CH_2_] and 1,2-dimethylallyl [H_2_C=C(CH_3_) ^•^CH(CH_3_)] radicals from H + isoprene [H_2_C=C(CH_3_)CH=CH_2_];^48^^•^C_5_H_5_NH and 4-C_5_H_6_^•^N from H + pyridine;^49^ 2,3-dihydropyrrol-2-yl (3-H^•^C_4_H_4_NH) and 2,3-dihydropyrrol-3-yl (2-H^•^C_4_H_4_NH) radicals from H + pyrrole (C_4_H_4_NH);^50^^•^ONH(OH) from H + nitrous acid (HONO);^51^ H^•^SO_2_, ^•^S(OH)_2_, HS(O)OH from H + SO/SO_2_;^52^ 2-hydrofuran-3-yl, 3-hydrofuran-2-yl, 2,3,4-trihydrofuran-5-yl, and 2,3,5-trihydrofuran-4-yl from H + furan;^53^ and H_2_C^•^NO from H + fulminic acid (HCNO)^54^ were recently observed by several groups. These H atom reactions were all studied using this UV/IR technique, demonstrating the generality of this approach, and none of these reactions could be studied at these temperatures in a noble gas matrix simply because the H atom is not mobile enough under such conditions.

However, when H atoms reacted with formamide HC(O)NH_2_, the H atom abstraction played an important role; the radical intermediate H_2_N^•^CO and the product of the second H atom abstraction, HNCO, were observed.^55^ A dual-cycle mechanism containing two sets of H atom abstraction and H atom addition reactions chemically links HC(O)NH_2_, H_2_N^•^CO, and HNCO together and might explain the nearly constant ratio of [HNCO]/[HC(O)NH_2_] in interstellar media. H atom abstraction reactions to produce radicals were also observed for methanol (CH_3_OH),^56^ ethanol (C_2_H_5_OH),^57^ methyl formate [HC(O)OCH_3_],^58^ acetamide [CH_3_C(O)NH_2_],^59^ acetic acid [CH_3_C(O)OH],^60^ glycine [NH_2_CH_2_C(O)OH],^61^ methyl amine [CH_3_NH_2_],^62^*N*-methyl formamide (NMF) [HC(O)NHCH_3_],^63^ and formaldoxime (H_2_CNOH);^54^ a recent review is available.^13^ From these experiments, we learned that in addition to the well-known H atom addition reactions, H atom abstraction reactions can also produce radicals and open up new channels for further reactions to form larger or more complex molecules. Furthermore, the coupling of H atom abstraction and H atom addition reactions can enable the uphill (endothermic) isomerization of molecules, as was observed in the conversion of the lower-energy *trans*-NMF to the higher-energy *cis*-NMF in darkness.^63^ The H atom addition can also induce fragmentation to form more stable smaller species, such as HNCO + CH_4_ and CH_2_NH + CO from H + *trans*-·C(O)NH(CH_3_).^63^ Similarly, CH_3_^•^CHOH was produced from H + C_2_H_5_OH.^64^

Recently, rich chemistry was observed for H + glycolaldehyde [HOCH_2_C(O)H, GA] in solid *p*-H_2_ by two groups.^65,66^ GA was detected in the interstellar medium (ISM); it is a primitive sugar-like molecule and a potential precursor for complex sugars. Radical intermediates HOCH_2_CH_2_^•^O (**1**), HOCH_2_^•^CHOH (**2**), HOCH_2_^•^CO (**3**), HO^•^CHC(O)H (**4**), and ^•^OCH_2_C(O)H (**5**) derived from GA might lead to the formation of glyceraldehyde, dihydroxyacetone, and ethylene glycol in the interstellar medium. The group in Taiwan recently conducted reactions of the H + *Cis*-*cis* conformer of GA (*Cc*-GA) in solid *p*-H_2_ at 3.2 K and identified IR spectra of radicals produced from H atom abstraction, *Cc*-HOCH_2_^•^CO (**3**) and *Cc*-HO^•^CHC(O)H (**4**), as well as the closed-shell species HOCHCO (**6**), produced via consecutive H atom abstraction reactions with GA. In addition, *cc*-HOCH_2_CH_2_^•^O (**1**) and ^•^CH_2_OH + H_2_CO were produced through the H atom addition and the H atom induced fragmentation channels, respectively. The reaction of H + GA indicates the multiple roles that GA might play in astronomical chemistry via its rich chemistry with four channels of three distinct types. Furthermore, the formation of *cc*-HO^•^CHC(O)H (**4**) and ^•^CH_2_OH + H_2_CO was found to be enhanced during near-IR irradiation of the sample as compared to reactions in darkness. Such a significant difference in the branching among different reaction channels, as was observed depending on whether the *p*-H_2_ matrix was being exposed to near-IR radiation or not, illustrates nicely that when studying such low-temperature chemical reactions, all possible energy sources must be characterized, as it appears that the H atoms in the near-IR irradiated *p*-H_2_ samples react differently from those in the *p*-H_2_ samples with no near-IR exposure.

Coupling the strongly exothermic reaction H + H → H_2_ (exothermicity ∼480 kJ mol^–1^) with potential endothermic reactions of guest species means that even endothermic reactions become feasible in the dark under interstellar conditions. This effect is similar to the coupling of ATP → ADP to drive originally endothermic reactions in biological systems; H atoms hence can be regarded as the ATP in the ISM. Because of the abundance of H atoms in astronomical environments, one must consider all possible H atom-assisted reactions, including the previously overlooked ones such as H atom abstraction, uphill isomerization, and H atom induced fragmentation, in astrochemical models.

On the other hand, because of the coupling of H atom abstraction and H atom addition reactions, H atoms might be catalytically converted to H_2_ by these astrochemical species; that means, mobile H atoms might be converted to molecular H_2_ without going through the direct H + H recombination reaction. These previously overlooked catalytic reactions might help to explain the observation of a smaller [H]/[H_2_] ratio in interstellar media as compared with the predicted ratio based on the recombination of H atoms in the gaseous phase and on dust grains.^67^

## Anomalous Temperature Effect

Typically, reaction rate coefficients increase with temperature; this Arrhenius temperature dependence is related to the barrier for the reaction. However, in solid *p*-H_2_, several H atom reactions have been found to occur only at temperatures lower than ∼3 K and to cease above that temperature. For example, the group in Laramie reported that the addition reaction H + N_2_O → *cis*-HNNO/*trans*-HNNO^41,68^ occurs below ∼2.4 K but ceases above 2.4 K. *Ab initio* calculations^69^ that take zero-point energy corrections into account predict that this H + N_2_O reaction has a barrier of 47 kJ mol^–1^, which means that this reaction can only take place via quantum-mechanical tunneling under these low-temperature conditions. In addition, H atom abstraction reactions conducted in solid *p*-H_2_ such as H + HC(O)OH → HOCO + H_2_,^70,71^ H + CH_3_OH → ^•^CH_2_OH + H_2_,^72^ and H + HC(O)OCH_3_ → ^•^C(O)OCH_3_/HC(O)O^•^CH_2_ (ref (58)) also showed similar anomalous temperature behaviors. All of these H atom abstraction reactions have sizable barriers, suggesting that this might be required to observe this nonintuitive low-temperature reactivity.

The reaction of H atoms with formic acid was one of the first reaction conducted in solid *p*-H_2_ that showed this anomalous low-temperature reaction kinetics.^70,71^ The reactivity of H atoms with formic acid has also been studied in noble-gas matrix-isolation experiments.^73^ H-addition products dominated the matrix-isolation studies conducted in a Kr matrix at 31 K, where the final product was identified^73^ as the simplest geminal diol radical *trans–cis*-HC(OH)_2_. This shows that the chemical reactivity is qualitatively different under these two contrasting low-temperature conditions. We believe that the differences stem from the different H atom diffusion mechanisms; in a noble-gas matrix, the chemistry is thermally induced such that the H atoms only start to diffuse above some threshold temperature, whereas in solid *p*-H_2_ the H atoms are mobile at all temperatures via a quantum-mechanical tunneling mechanism. Thus, this anomalous low-temperature chemistry cannot be studied in a noble-gas matrix and, therefore, is likely related to the details of H atom quantum diffusion in solid *p*-H_2_.

We point out that this anomalous low-temperature reaction kinetics is different from what has been observed in several gas-phase reactions in which the reaction accelerates very rapidly at very low temperatures.^74^ For gas-phase hydrogen abstraction reactions of OH with organic compounds containing alcohol, ether, carbonyl, and ester functional groups, the rate coefficients at very low temperatures can be up to 1000 times larger than those at room temperature, despite the barrier to products.^74^ This anti-Arrhenius behavior has been explained by the formation of a weakly bound hydrogen-bonded complex of the reactants, such that at low temperatures the lifetime of the complex against redissociation back to reactants becomes much longer, and hence the probability of quantum-mechanical tunneling under the reaction barrier to form products becomes much larger. A comparable mechanism could be operative for H atoms in solid *p*-H_2_ where only at the lowest temperatures the H atom gets trapped in a weakly bound complex with a potential reactant, such that there is an increased probability of reaction. However, for all of the reactions conducted in solid *p*-H_2_ that have shown this anomalous temperature behavior, we do not measure an upturn in the reaction rate coefficient similar to what has been observed under gas-phase conditions.

**Figure 3:**
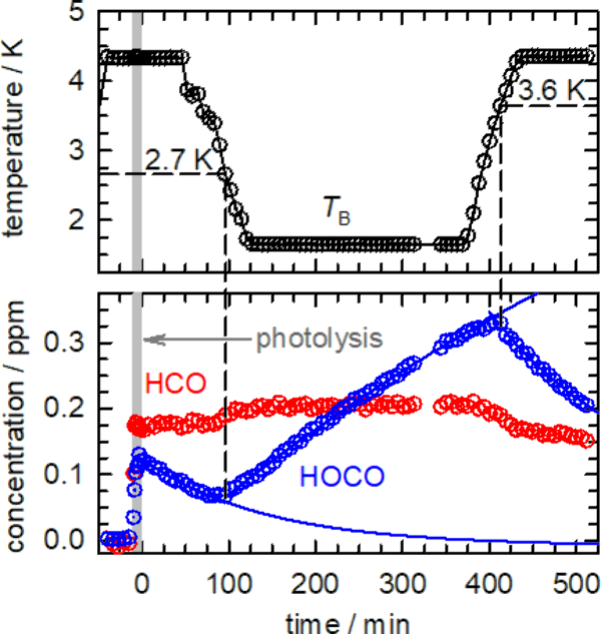
Kinetic plots for the 193 nm photolysis (10 min, 18 mW cm^–2^) induced reaction study conducted on a HC(O)OH/*p*-H_2_ sample as a function of temperature. The top graph shows the temperature of sample *T*_B_, and the bottom graph shows the measured HOCO (blue circles) and HCO (red circles) concentrations. This experiment shows that for a sample photolyzed at 4.3 K, the reaction that produces HOCO after photolysis only starts the first time the temperature is lowered below ∼2.7 K. The kinetics of this reaction again qualitatively changes at later times when the temperature is raised above 3.6 K. Partially reproduced from ref (70). Copyright 2014 American Chemical Society.

Detailed kinetic studies conducted by Mutunga et al. on the H + N_2_O reaction^41^ in solid *p*-H_2_ showed that the rate coefficient does not increase at lower temperatures, but rather the reaction mechanism switches abruptly such that H atoms do not react with N_2_O at temperatures above 3 K. Shown in Figure 3 are kinetic plots for the H atom reaction study with formic acid in which the temperature is varied during the reaction. As seen in Figure 3, the reaction is initiated at 4.3 K by partial *in situ* photodissociation of formic acid at 193 nm to generate H atoms, and the HOCO that is generated during the photolysis step slowly decays after the photolysis laser is stopped while the temperature is maintained at 4.3 K. There are five open photodissociation channels^75^ for formic acid at 193 nm, and the radical channels produce HCO, OH, HOCO, and H atoms. However, once the temperature is reduced to ∼2.7 K, all of a sudden the HOCO concentration begins to grow due to H atom abstraction reactions with formic acid to produce HOCO and H_2_. Then at later stages of the experiment, the temperature is again increased and, when the temperature reaches ∼3.6 K, the kinetics again switch to HOCO decay. However, when we measure the kinetics as a function of temperature, we do not observe an increased rate coefficient at lower temperatures; the rate coefficient is essentially temperature-independent over the temperature range 1.5–4.3 K. This lack of the upturn in the rate coefficients with a decrease in temperature led us to speculate that the reaction mechanism responsible for the observed kinetics in solid *p*-H_2_ is different from what is observed in some gas-phase reactions in which a long-lived prereactive complex speeds up the kinetics, but this idea might be premature. In general, gas-phase and condensed-phase reaction kinetics are very different, such that participation of a long-lived prereaction complex in the reaction mechanism could have very different effects on the reaction kinetics under different reaction conditions. The group in Laramie is conducting detailed kinetic studies on H atom reactions with O_2_, CO, and NO in which these anomalous temperature effects have not been observed. Indeed, the reactions that show anomalous low-temperature reaction kinetics involve reactions with appreciable barriers and involve reactant molecules that contain functional groups such as carbonyls, alcohols, and esters. Thus, the anomalous low-temperature reaction kinetics may be related to preferred trapping sites adjacent to the coreactant that are only populated at low temperatures. What is really needed at this point is computational studies that can treat H atom quantum-diffusion in chemically doped *p*-H_2_ solids, such as the path integral molecular dynamics simulations conducted by Voth and co-workers in the early 2000s.^76^

## Heavy-Atom Diffusion

As discussed previously, H atom diffusion in solid *p*-H_2_ has been known for over 50 years, but the diffusion of heavier species remains largely unexplored.^77^ However, two studies have provided preliminary information about molecular diffusion in solid *p*-H_2_. One group studied HF diffusion in solid *p*-H_2_ at ∼4 K by monitoring the temporal decay of the HF monomer absorption due to dimerization using infrared absorption spectroscopy.^78^ These researchers hypothesize that because the activation energy for the dimerization reaction, HF + HF → (HF)_2_, must be small or near zero, this reaction is diffusion-controlled in the solid phase, so that the rate coefficient *k* for dimerization is proportional to the diffusion coefficient *D* of HF in solid *p*-H_2_. They found that the decay in the HF monomer absorption peaks was well fit by second-order rate equations, which they used to extract the rate coefficient for the growth of HF clusters (dimer) as a function of time after deposition. In this study, they showed that the diffusion rates were affected by the sample temperature, the initial HF concentration, and annealing the sample. They also showed that the infrared light from their FTIR spectrometer speeds up the diffusion, presumably by excitation of rovibrational motion of HF or *p*-H_2_.

More recently, Momose and co-workers^79^ studied the diffusion of H_2_O molecules in solid *p*-H_2_ using a similar approach. By monitoring the temporal decay in H_2_O monomer absorption features and the growth of cluster peaks (dimer, trimer, and tetramer), they could fit their data to models of diffusion-controlled nucleation. Similar to the studies of HF diffusion,^78^ they found that the diffusion rate is inversely proportional to the concentration of water molecules due to a lowering of the periodicity of the solid. It is known that the efficiency of a quantum-tunneling process is strongly enhanced when the energy levels between the initial and final states of the tunneling process are nearly resonant. Kagan and Leggett^80^ pointed out that the rate of quantum diffusion therefore substantially decreases with a lowering of the periodicity of the solid due to poor resonance. In the water diffusion experiments,^79^ it is the H_2_O molecule itself that lowers the periodicity of the lattice, and thus, quantum diffusion is slower in more concentrated samples. By measuring how the diffusion coefficient explicitly depends on the water concentration, they found evidence that there might be correlated motion of the water molecules, a signature of quantum diffusion.^79^ These researchers also showed, through detailed modeling of the kinetics, that both the water monomers and dimers migrate through solid *p*-H_2_. However, the group in Laramie also point out that both Br atoms^81^ and Li atoms^82^ are thought to only diffuse in solid *p*-H_2_ at elevated temperatures of 4.3–4.4 K, which is interpreted as thermal diffusion. Adding to the confusion, the authors of the HF diffusion paper^78^ stated that H_2_O does not diffuse in solid *p*-H_2_ at 3.6 K, nor does CO, but the later experiments of Momose and co-workers^79^ showed that H_2_O does quantum-diffuse in solid *p*-H_2_. Kumada et al. reported^83^ that Ne atoms cannot diffuse in solid *p*-H_2_ at 4.2 K despite their size and mass being comparable to those of HF. Thus, there is considerable uncertainty in whether other heavy atoms and molecules can diffuse in solid *p*-H_2_. Is there something special about HF and H_2_O that permits facile quantum diffusion? Some conflicting results are likely related to the various experimental conditions used to look for quantum diffusion, where lower dopant concentrations and lower temperatures are advantageous to observe quantum diffusion.

**Figure 4:**
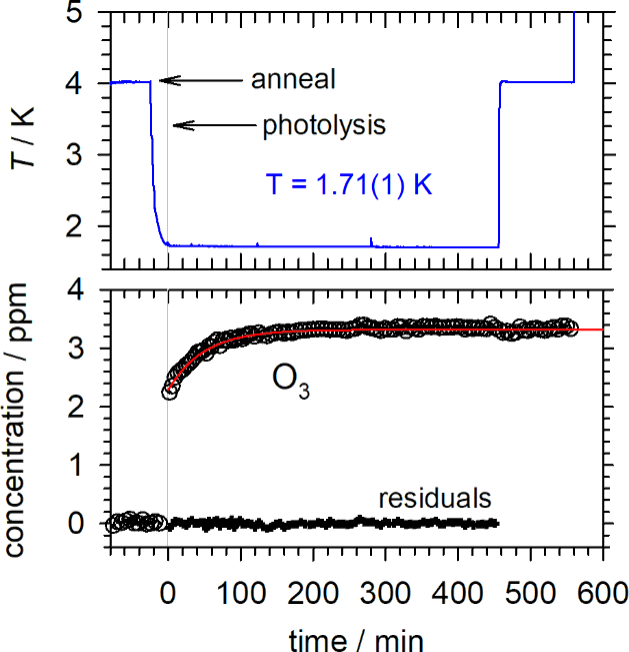
Ozone growth kinetics for an annealed O_2_/*p*-H_2_ sample recorded at 1.71(1) K after a 0.5 min 193 nm photolysis exposure. The O_3_ concentration is plotted as black circles, and the red line is the result of a least-squares fit of the data to a first-order kinetics equation. Partially reproduced from ref (74). Copyright 2023 American Chemical Society.

The group in Laramie recently demonstrated that oxygen atoms can also quantum diffuse through solid *p*-H_2_ at appreciable rates.^84^ Experiments on the 193 nm *in situ* photolysis of O_2_ trapped in solid *p*-H_2_ were performed to measure the diffusion-controlled kinetics of the O(^3^P) + O_2_ → O_3_ reaction via infrared spectroscopy of the O_3_ reaction product. Short-term exposure of an O_2_-doped *p*-H_2_ solid to 193 nm radiation produces O atoms in their ground electronic state O(^3^P); the 193 nm photon energy is not capable of producing O(^1^D).^85^ Therefore, once the O(^3^P) atom becomes equilibrated at liquid helium temperatures, it cannot react with the *p*-H_2_ host even by quantum-mechanical tunneling because the O(^3^P) + H_2_ → OH + H reaction is endothermic (Δ*H* = +944 cm^–1^).^86^ However, during *in situ* photolysis the nascent O(^3^P) atoms are generated with a maximum translational energy of 62.6 kJ mol^–1^, which is greater than the calculated barrier, 55.2 kJ mol^–1^, for the reaction, so the possibility of reaction seems high. Yet, the light mass of the *p*-H_2_ host means that the collision energy in the center-of-mass frame between a fast-moving O atom and a stationary H_2_ molecule is very low, and thus, short-term 193 nm photolysis can be used to generate O atoms in solid *p*-H_2_ with little evidence of O(^3^P) reactions. After photolysis, continued growth in the O_3_ concentration was observed for up to 600 min due to the O(^3^P)-atom quantum diffusion. A representative trace of the kinetics of the O_3_ is shown in Figure 4 for an annealed O_2_/*p*-H_2_ sample. The O(^3^P) + O_2_ reaction is barrierless,^87,88^ and therefore, we expect that the reaction is diffusion-controlled so that by measuring the reaction kinetics we can estimate the diffusion coefficient for O(^3^P) atoms in solid *p*-H_2_. The reaction rates and O_3_ yields are strongly affected by the *p*-H_2_ crystal morphology, where in as-deposited solids that contain mixed crystal structures and more defects, the rate coefficient is 2× slower than in annealed solids. As expected for quantum diffusion-controlled reaction kinetics for a barrierless reaction, increasing the temperature from 1.7 to 4.0 K did not increase the rate coefficient significantly.

The surprising result from the O(^3^P)-atom reaction studies was that the average O(^3^P)-atom diffusion coefficient is comparable to the best literature values for H atom quantum diffusion in solid *p*-H_2_.^84^ Specifically, the diffusion coefficient extracted from the O(^3^P) atom reaction at 1.7 K for annealed *p*-H_2_ solids is *D* = 7.6(2) × 10^–17^ cm^2^ s^–1^ and the diffusion coefficient for H atom quantum diffusion is *D* = 2.7(3) × 10^–16^ and *D* = 1.7(2) × 10^–16^ for *n*-H_2_ and *p*-H_2_, respectively, at 4.2 K.^84^ The previous measurements of the diffusion coefficient for H atoms in solid *n*-H_2_ and *p*-H_2_, respectively, were based on measuring the reaction kinetics of H atom recombination (H + H → H_2_). Measuring the diffusion coefficient indirectly in this way may introduce systematic errors due to the nature of quantum diffusion. When the distance between the recombining H atoms is small, the major potential inhomogeneity for quantum tunneling is associated with the interaction of the pairing particles.^83^ This can cause the final stages of recombination to be very slow or not to occur at all because the tunneling bandwidth is much smaller than the energy level mismatch caused by the interacting particles. This possibility is pointed out in the paper that measured the H atom diffusion coefficient where they observed an “absence” of recombination in heavily enriched *p*-H_2_ solids.^89^ However, in more recent measurements^90^ on H_2_ thin films at temperatures below 1 K, the H atom recombination kinetics can be distinguished from spatial diffusion driven by a concentration gradient, allowing the researchers to test for this systematic bias. These researchers showed that for both *n*-H_2_ and *p*-H_2_ thin films, the H atom diffusion coefficient measured for pure spatial diffusion is 1 to 2 orders of magnitude larger than the diffusion coefficient extracted from H atom recombination.^90^ This is consistent with our picture of quantum diffusion, where the diffusion process is the fastest when the H atom propagates through a periodic matrix potential via resonant tunneling. Therefore, more diffusion studies in solid *p*-H_2_ involving other atoms and molecules are needed to better characterize the conditions that lead to fast quantum diffusion in solid *p*-H_2_.

## Electronic Transitions

Investigations of the electronic transitions in *p*-H_2_ are scarce. The best example to show the advantages associated with the unique properties of *p*-H_2_, the softness of the matrix, is the Rydberg transitions of NO.^91^ The electronic transition from the ground *X*^2^Π state to the Rydberg state *A*^2^Σ^+^ is known to produce a bubble-like electronic cloud. For noble-gas matrix hosts, the rigidity of the solid limits the expansion of the electronic bubble upon excitation, hence inducing a large blue shift for this transition. In the gaseous phase, the NO (*A–X*) transition origin was reported to be 44080.5 cm^–1^,^92^ whereas those in solid Ne and Ar were reported to be 45536 and 46377 cm^–1^,^93,94^ respectively, with blue matrix shifts greater than 1450 cm^–1^. In contrast, the NO (*A–X*) transition origin in *p*-H_2_ was observed at 44105 ± 20 cm^–1^, with a blue shift of only ∼25 cm^–1^.

On the basis of the electronic spectroscopy of NO in solid *p*-H_2_, one would expect small and less-divergent matrix shifts for electronic transitions of species of various types in solid *p*-H_2_. If this would be true, because producing protonated, cationic, and hydrogenated species in *p*-H_2_ is much easier than producing these species in the gaseous phase or in noble-gas matrices, as discussed previously, one would be able to identify possible carriers of diffuse interstellar bands (DIBs) by comparison of electronic spectra of potential molecules trapped in solid *p*-H_2_ with observed DIBs, taking the expected matrix shift and uncertainties into consideration as a preliminary test. However, so far, only fluorescence excitation spectra and dispersed fluorescence of 1-hydronaphthyl radical (1-C_10_H_9_),^95^ sumanene (C_21_H_12_),^96^ and *peri*-hexabenzo-coronene (C_42_H_18_)^97^ have been reported; a lot of data need to be collected before any conclusion can be drawn.

Another advantage of investigating electronic transitions with the matrix-isolation technique is that the transition origin can be unambiguously identified because vibrational relaxation is rapid in matrices, and typically emission comes only from the vibrational ground state and often from the lowest electronic state of the same spin multiplicity. In the case of ovalene, a literature report indicated a transition origin of S_1_–S_0_ at 466.22 nm (21449 cm^–1^) in the gaseous phase,^98^ but experiments in solid *p*-H_2_ indicated that the band was misassigned by one vibrational quantum; the origin should be ∼473.1 nm (21135 cm^–1^) in the gaseous phase and was observed at 475.1 nm (21050 cm^–1^) in *p*-H_2_ with a lifetime of 1.5 μs.^99^ Furthermore, according to the spectral pattern of electronic emission (corresponding to dispersed fluorescence) and absorption (corresponding to fluorescence excitation) predicted with the Franck–Condon Herzberg–Teller approach, the reported transition should be the *S*_2_(*B*_3u_)–*S*_0_(*A*_g_) rather than the *S*_1_(*B*_2u_)–*S*_0_(*A*_g_) transition. This example also demonstrates the advantage of using *p*-H_2_ for the correct spectral assignments.

**Figure 5:**
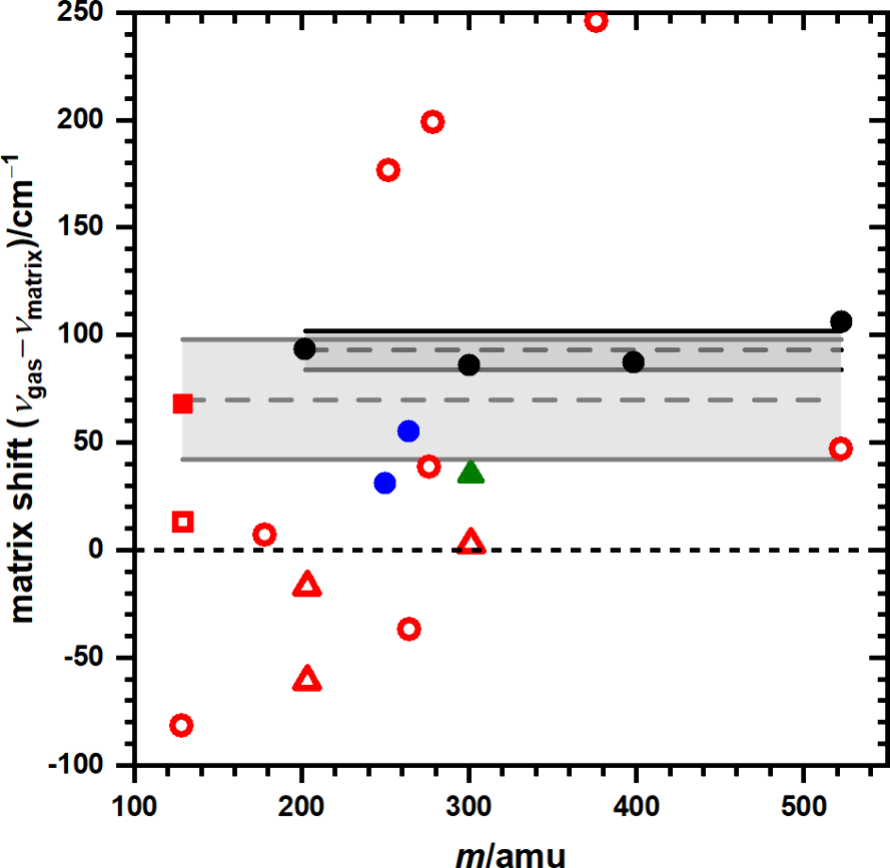
Matrix shifts for species in solid *p*-H_2_ and Ne as a function of mass *m*. Solid symbols: species in solid *p*-H_2_; open symbols: species in solid Ne. Symbols: circles: PAH (black for planar PAH and blue for nonplanar PAH in solid *p*-H_2_); square: hydrogenated PAH; triangles: protonated PAH. The average matrix shift of planar PAH in *p*-H_2_ (black solid circles) is 93 ± 9 cm^–1^ and that of all species in *p*-H_2_ (solid symbols) is 70 ± 28 cm^–1^, as indicated with dashed lines and gray regions representing listed uncertainties as one standard deviation in fitting.

The group in Taiwan has recorded the dispersed fluorescence and fluorescence excitation spectra of several PAH and a few hydrogenated and protonated PAH, of which the gaseous transition origins have been reported. Figure 5 shows the matrix shifts (including unpublished results) of several PAH and their derivatives as a function of mass *m*; solid symbols represent values for solid *p*-H_2_ and open symbols represent literature values in the Ne matrix. Among them, circles indicate PAH (black for planar PAH and blue for nonplanar PAH in solid *p*-H_2_), squares indicate hydrogenated PAH, and triangles indicate protonated PAH. The average matrix shift, ν_gas_ – ν_matrix_, of planar PAH in solid *p*-H_2_ is 93 ± 9 cm^–1^, whereas that for all species in solid *p*-H_2_ is 70 ± 28 cm^–1^; listed errors represent one standard deviation in fitting. As compared with solid Ne, even though the matrix shifts of a majority of species in *p*-H_2_ are greater than those in Ne, they show shifts only to the red and with small variations.

The limited data showing that the matrix shifts for electronic transitions of PAH and their derivatives in *p*-H_2_ are always red-shifted and with small variations look promising. If the average value can be reliably applied, then the wavenumbers of absorption bands observed in solid *p*-H_2_ can be corrected to yield the estimated gas-phase values with uncertainties <60 cm^–1^ at the 95% confidence level; for a band near 600 nm, this uncertainty corresponds to ±2.2 nm. The additional advantage is that many protonated and hydrogenated PAH can be readily produced only in solid *p*-H_2_, not in noble-gas matrices.

## Summary and Future Perspectives

In summary, several advantages of using *p*-H_2_ as a host for matrix isolation as compared with traditional noble-gas hosts have been demonstrated. These include the production of free radicals or unstable species via *in situ* photoirradiation; the production of protonated and hydrogenated species by using electron bombardment; efficient low-temperature H atom reactions in complete darkness via quantum diffusion of the H atoms; the anomalous temperature effects in reactions; the diffusion of heavy atoms or molecules; and the less divergent, consistently red matrix shifts for most electronic transitions.

Photoirradiation of guest species isolated in solid *p*-H_2_, either via direct photodissociation or using photoinduced bimolecular reactions, is an efficient and convenient way to produce free radicals for spectral characterization; in most cases, this method is not possible in noble-gas matrices due to the cage effect. The infrared and electronic spectra of many radicals await to be explored using *p*-H_2_ matrix-isolation spectroscopy. Furthermore, with clever design, more complicated experimental procedures such as irradiation–reaction–irradiation may be applied to generate radicals that are challenging to produce with irradiation in a single step. The “soft” environment of the *p*-H_2_ lattice is also conducive to the study of the relative motion in molecular complexes such as the bevel-gear-type motion in C_6_H_6_Br; future investigations on this type of motion and their associated chemistry such as stereoselectivity, chirality, or photochemistry become possible.

The production of protonated and monohydrogenated species using electron bombardment of a *p*-H_2_ matrix is straightforward and unique. The band positions in the IR spectra of protonated coronene and protonated corannulene seem to be approaching those in the interstellar UIR bands, but even larger protonated PAH awaits exploration to have improved matches. Unfortunately, the availability of these large amounts of PAH is rather limited. Perhaps a top-down method using graphene or C_60_ might give some hints on this puzzle. Furthermore, a comparison of the UV-induced IR emission with IR absorption spectra is necessary to ensure that assigning UIR emission bands according to IR absorption is practical. Even though monohydrogenated PAH appear to be unrelated to the UIR emission, they may play some roles in the interstellar catalytic H_2_ formation. The photochemistry and additional hydrogen reactions of PAH are required for further investigations.

The method for inducing H atom reactions in darkness using Cl_2_ as the initiator is very efficient and distinct from the H atom bombardment experiments in which the matrix or ice sample is bombarded with large amounts of H atoms. In the latter experiments, the large number of H atoms might induce secondary reactions such that reaction intermediates are typically difficult to identify using this approach. In *p*-H_2_ matrix experiments without input of energy (in darkness), the temporal evolution of each species (reactants and products) is followed in real time and hence provides valuable information to deduce the reaction mechanism. Although the *p*-H_2_ environment is obviously different from interstellar ices, investigations of H atom reactions in solid *p*-H_2_ provide a fundamental understanding of the *possible* low-temperature reaction mechanisms that might lead to new synthetic routes. As *p*-H_2_ matrix-isolation research has demonstrated, H atom abstraction and H-atom-induced fragmentation likely play more important roles in astrochemical environments than previously thought. Furthermore, by coupling H atom abstraction and addition reactions, two interstellar species might be chemically linked and form a quasi-equilibrium; transformation to an isomer with a higher energy is also possible. So far, H atom reactions of only a limited number of amides and small amino acids have been investigated. Investigations on the reactions of H atoms with larger amino acids, nucleobases, and other biologically related species might provide new insights into their reactions. The difference in the branching among various channels of H atom reactions between periods with near-IR irradiation and that in darkness, as was observed in the H + glycolaldehyde system, deserves further experimental and theoretical investigations.

The method of using UV/IR irradiation of a *p*-H_2_ matrix containing trace Cl_2_ to perform H atom reactions has one major disadvantage; the available H atoms for reactions in complete darkness are limited to the remnant H atoms that remain unreacted after near-IR irradiation, which places a constraint on the extent of H atom reactions that can proceed in darkness. Recently, a new method employing UV photolysis of a *p*-H_2_ matrix containing trace H_2_O_2_ was developed; the OH thus produced upon photolysis can react with H_2_ via tunneling to produce H_2_O and H atoms without the need for near-IR irradiation. This method allows for continuous generation of H atoms in darkness after UV photolysis of H_2_O_2_. Preliminary experiments on a UV-irradiated H_2_O_2_/CH_3_NH_2_/*p*-H_2_ matrix indicated that the H atom reactions can proceed further in this system, so that a significant amount of CH_2_NH and HCN was produced, as compared with those from the UV/IR-irradiated Cl_2_/CH_3_NH_2_/*p*-H_2_ matrix, indicating that abstraction of more H atoms in CH_3_NH_2_ is possible using this method. The disadvantages of this method are that a shorter wavelength is needed to dissociate H_2_O_2_, the byproduct H_2_O might interfere with the measured chemistry, and OH might compete with H atoms for reactions. These two methods are, hence, complementary to each other and can be used to study the same reaction. In view of the limitations and possible interference in these two methods, further new methods with high efficiency and less interference are waiting to be developed.

The anomalous temperature dependence observed for several H atom abstraction reactions conducted in solid *p*-H_2_ points to the complexity of the low-temperature solid-state reaction kinetics. Using solid *p*-H_2_ matrix isolation to study low-temperature chemistry allows the potential energy landscapes and reaction mechanisms to be investigated at unprecedented levels of detail. It is the mobility of the H atom in solid *p*-H_2_ that makes this all possible, and thus, the details of H atom quantum diffusion in solid *p*-H_2_ should continue to be studied to support our conviction that solid *p*-H_2_ can be used as a model system to probe the reactions occurring in and on interstellar ices. As discussed in this Perspective, the greater reactivity at lower temperatures is believed to be related to the pathway of H atom quantum diffusion through the solid and not that the reaction rate coefficient is increased at lower temperatures. The facile quantum diffusion of H atoms through solid *p*-H_2_ means that low-temperature bimolecular reactions can be studied in the laboratory on multiple-hour time scales, and the analogous studies in interstellar ice analogues or noble gas matrices is simply not possible because the H atom diffusion rates are much slower and thermally activated. While mass transport in solid *p*-H_2_ is very different from that in any other cryogenic matrix host, the chemical reactivity should be comparable. This is why the studies of the O atom reactions are also exciting because O atom chemistry can also be explored using *p*-H_2_ matrix isolation if efficient ways of O atom generation are developed.

Even though the present data support a consistently red and less-divergent matrix shift for electronic transitions in solid *p*-H_2_, many more data need to be collected before one can make such a conclusion. The problem that one faces is that limited data are available for PAH and PAH-derivatives in the gaseous phase or trapped in solid Ne for comparison, because investigations on the electronic transitions of these species are challenging. In contrast, producing these PAH-derivatives using *p*-H_2_ is less challenging, so their electronic transitions may be investigated without much difficulty. Finding a promising candidate of DIB would provide a big boost for the investigation of electronic transitions in solid *p*-H_2_. For proper assignments of the observed bands, quantum-chemically predicted spectra based on Franck–Condon Hertzberg–Teller or even higher-level calculations are indispensable. However, for some “difficult” electronically excited states, accurate predictions using the standard TD-DFT method are challenging. More sophisticated methods are needed to tackle these “difficult” states in order to support the assignments of observed bands.
